# Possible detrimental effects of postponed intubation during interhospital transfer of severely brain injured patients: retrospective analysis of the Traumaregister DGU^®^

**DOI:** 10.1007/s00068-026-03082-y

**Published:** 2026-02-02

**Authors:** Ferdinand C. Wagner, Daniel Witry, Rolf Lefering, Christoph Scholz, Hagen Schmal, Jörg Bayer

**Affiliations:** 1 Department of Orthopaedic and Trauma Surgery, Medical Center, Faculty of Medicine, University of Freiburg, Hugstetter Straße 55, 79106 Freiburg, Germany; 2 Department of Trauma and Orthopaedic Surgery, Schwarzwald-Baar Hospital, Klinikstraße 11, Villingen-Schwenningen, 78052 Germany; 3 Institute for Research in Operative Medicine (IFOM), Faculty of Health, University Witten/Herdecke, Ostmerheimer Straße 200, Cologne, 51109 Germany; 4 Department of Neurosurgery, Medical Center, Faculty of Medicine, University of Freiburg, Breisacher Straße 64, Freiburg, 79106 Germany; 5 Department of Orthopedic Surgery, University Hospital Odense, Sdr. Boulevard 29, Odense, 5000 Denmark

**Keywords:** Traumatic brain injury, Airway management, Emergency department management, Neurotrauma center

## Abstract

**Purpose:**

Prehospitally, the paradigm of obliged intubation in traumatic brain injured (TBI) patients with a reduced Glasgow Coma Scale (GCS) < 9 has been debated. Many patients with severe TBI need interhospital transfer to definitive care, so we sought to elucidate possible disadvantages for patients with a reduced level of consciousness where intubation prior transportation was withheld.

**Methods:**

Transferred patients with at least serious blunt injury to the head (Abbreviated Injury Scale_Head_ ≥3) were analyzed. In depth analysis was conducted in patients with GCS 4–9. We applied multivariate regression analysis to search for relevant variables for mortality differences and scrutinized patients who needed immediate intubation upon arrival in the emergency room (ER ITN). In this context a “postponed” intubation refers to an intubation performed upon arrival at the receiving hospital rather than prior to transfer.

**Results:**

Comparing spontaneously breathing versus already intubated patients (ITN) at admission we did not find statistically significant differences in mortality (33.2% vs. 20.4%; *p* = 0.067), multiple organ failure (MOF) (37.1% vs. 34%; *p* = 0.667) or sepsis rates (13.6% vs. 4.2%; *p* = 0.069). Multiple regression analysis for mortality revealed only age > 70 years, coagulopathy and AIS_Head_ ≥ 5 as significantly associated independent variables. But, comparing patients requiring intubation in the emergency room upon admission (ER ITN) to patients already intubated prior transportation (ITN), we detected significant differences in MOF (53.7% vs. 34%; *p* = 0.025), sepsis (20.5% vs. 4.2%; *p* = 0.011) and mortality rate (38.1% vs. 20.4%; *p* = 0.028).

**Conclusion:**

Our results may suggest an inferior outcome when intubation in patients with GCS 4–9 is needed during admission at the receiving hospital. Further research is warranted to scrutinize optimal airway management for interhospital transfer of TBI patients with reduced GCS.

## Introduction

Traumatic brain injury (TBI) constitutes one of the leading causes of injury-related disability and mortality worldwide [1]. It results from external mechanical forces applied to the head, leading to primary injury at the moment of impact and often followed by secondary injury as a consequence of physiological responses [2]. TBI is a medical emergency, requiring prompt management to prevent secondary brain damage and improve patient outcomes. In the acute setting, the initial management of moderate-to-severe TBI is crucial to stabilize the patient, reduce secondary injury, and maintain homeostasis. This often involves advanced airway management and resuscitation to maintain hemodynamic stability [3]. Endotracheal intubation is considered the gold standard in definitive airway protection, and patients with severe TBI are frequently intubated in the field by prehospital emergency medical personnel [4]. Recommendations exist for anesthesia, endotracheal intubation and mechanical ventilation in patients with severe traumatic brain injury defined by Glasgow Coma Scale (GCS) < 9 [5–7]. However, well-founded evidence to support this widespread practice is scarce. While prehospital intubation should theoretically be beneficial to ensure airway protection and to prevent hypoxemia [8], the procedure also involves risks that may lead to adverse outcomes. The role of endotracheal intubation in the prehospital setting for patients with TBI has historically been an area of controversy. Multiple studies of varying quality have shown conflicting outcomes for TBI patients undergoing prehospital intubation [9] resulting from adverse effects e.g. hypoxemia due to prolonged intubation attempts, increases in intracranial pressure during laryngoscopy, hemodynamic effects of anesthetic drugs, hypo- or hypercapnia due to inappropriate ventilation following endotracheal intubation, and delayed transport to definitive care [4, 10, 11]. 

The effect of prehospital intubation on mortality might depend on emergency medical clinician experience, and it seems prudent to involve prehospital personnel well proficient in prehospital intubation whenever intubation is potentially required [12]. Yet, more recent reports in adult trauma patients with blunt head trauma suggest that routine intubation may be harmful for some patients with GCS ≤ 8 as it may be associated with an increased risk of complications and mortality [13, 14]. 

TBI patients initially admitted to acute care trauma hospitals (ACTH) may receive definitive care on site or undergo interhospital transfer to a neurotrauma center (NTC) for access to neurosurgery or neurocritical care [15, 16]. As much as 40–50% of all patients with moderate-to-severe TBI are primarily admitted to non-neurosurgical ACTHs, making interhospital communication and transfer to NTCs essential for patients who require neurosurgery or neurocritical care [17, 18]. Information about injury type and severity, as indicated by head computed tomography (CT) findings, neurological deficits, and Glasgow Coma Scale (GCS) scores, are decisive determinants for transfer decisions because they define indications for emergency neurosurgery or neurointensive care [19]. Careful secondary triage at admission identifies patients anticipated to benefit from specialized care and transfer [17], but transfer of patients with a brain injury is potentially hazardous if poorly executed and long-term neurological outcome may be adversely affected [20]. As far as definitive airway management is considered, recommendations exist to intubate patients with GCS ≤ 8 prior to transportation into a NTC [20]. 

Because of conflicting evidence for intubation of patients suffering from severe TBI with GCS ≤ 8 in the prehospital setting versus treatment recommendations in-hospital before transferring patients to a NTC we sought to evaluate the effect on outcome in transferred TBI patients with depressed level of consciousness and no intubation prior transport to a NTC.

## Methods

The TraumaRegister DGU^®^ (TR-DGU) of the German Trauma Society (Deutsche Gesellschaft für Unfallchirurgie, DGU) was founded in 1993 and represents a multi-center database containing pseudonymized and standardized documentation of severely injured patients. Data are collected prospectively in 4 consecutive phases: (A) pre-hospital phase, (B) trauma room and initial surgery, (C) intensive care unit and (D) discharge. The documentation includes detailed information on demographics, injury patterns, comorbidities, pre-hospital and clinical management, intensive care course, key laboratory findings including transfusion data, and outcome. The inclusion criterion is admission to hospital via the trauma room with subsequent monitoring in intensive or intermediate care, or arrival at hospital with vital signs and death before admission to intensive care unit (ICU).

The infrastructure for documentation, data management and data analysis are provided by the AUC - Academy for Trauma Surgery (AUC - Akademie der Unfallchirurgie GmbH), which is affiliated to the German Trauma Society. The scientific leadership is provided by the Committee on Emergency Medicine, Intensive Care and Trauma Management (Sektion NIS) of the German Trauma Society. Participating clinics enter their pseudonymized data into a central database via a web-based application. Scientific analyses are approved according to a peer review process defined in the publication guidelines of the TR-DGU. The participating clinics are mainly located in Germany (90%), but an increasing number of clinics from other countries are also contributing data (currently from Austria, Belgium, China, Finland, Luxembourg, Slovenia, Switzerland, the Netherlands and the United Arab Emirates). Currently, about 38,000 cases from almost 700 clinics are added to the database each year. Participation in the TR-DGU is on a voluntary basis. It is mandatory for TraumaNetzwerk DGU^®^ clinics to enter at least one basic data set for quality assurance purposes.

The study strictly adheres to the publication guidelines of the TR-DGU and is registered under the TR-DGU project ID 2022-026. The research project has been approved by the local ethics committee (University of Freiburg, 24-1587-S1-retro) and informed consent was waived.

### Patients

This retrospective multicenter observational study used the TR-DGU registry data to extract information on multiply injured adult patients after inter-hospital transfer. The data were filtered based on strict inclusion and exclusion criteria, with a serious head injury being the determining factor. Patient selection was based on the following *inclusion criteria*:

1. Online standard documentation from German Level 1 and Level 2 trauma centers.
2. Admission between 01-01-2012 and 31-12-2023.
3. Age ≥ 18 years.
4. Serious blunt head injury defined as an abbreviated injury scale (AIS)_Head_ ≥ 3.
5. Patients being transferred into the documenting hospitals.

Patients were *excluded* from further analysis with:

1. Missing information on GCS and/or airway status (intubation, management) in the emergency room (ER) on admission.
2. Penetrating injury.
3. One or more serious injuries to other body regions than the head (AIS > 3).

**Fig. 1:**
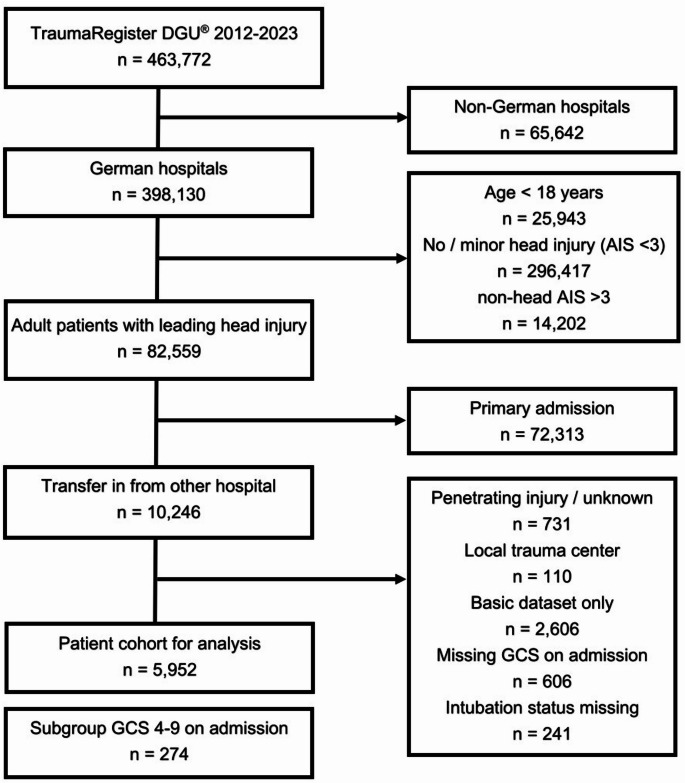
Study flow diagram illustrating the selection of patients

Patient selection and flow diagram are depicted in Fig. 1.

Injuries were graded according to the 2005, update 2008, version of the Abbreviated Injury Scale (AIS) [21], and the Injury Severity Score (ISS) was calculated as described earlier by Baker et al. [22] Polytrauma patients and coagulopathy were defined according to the “Berlin definition” [23]. 

The initial analysis comprised datasets of all admitted patients and were divided into already intubated patients at admission (ITN) and spontaneously breathing patients (no-ITN). After statistically comparing these two groups epidemiologically and for mortality differences by multivariate regression analysis, we defined another patient subgroup for further analysis. Because patients who required intubation immediately after admission to the ER might have had the indication for intubation at the transferring hospital already, we used this characteristic to define this specific patient subgroup (ER ITN). By comparing this group (ER ITN) epidemiologically to already intubated patients at admission (ITN) we sought to find possible disadvantages in delayed intubation. In this context a “postponed” intubation refers to an intubation performed upon arrival at the receiving hospital rather than prior to transfer.

### Statistical analysis

Continuous variables are shown as mean with standard deviation (SD) or median and interquartile range (IQR), depending on the distribution, and incidence rates are expressed as percentages. Differences in the study population were compared with the χ^2^-test for categorical variables and the Mann-Whitney U-test for continuous variables, respectively.

Statistical analysis of outcome was performed using multiple logistic regression analysis (MLR), with hospital mortality as dependent variable. Only patients with GCS 4–9 at admission were included. The independent variables consisted of age, American Society of Anesthesiologists (ASA) physical status (ASA 3/4) before the injury, AIS_Head_, relevant (AIS 2–3) accompanying injuries to other (non-head) body regions, coagulopathy (Quick’ value ≤ 60%, or PTT ≥ 40 s, or INR ≥ 1.4), intubated prior to admission. Results are reported as odds ratios (OR) with 95% confidence intervals (CI). Nagelkerke’s R² was used to describe the predictive power of the model. A p-value < 0.05 is considered statistically significant.

Statistical analysis was conducted using SPSS (Version 29.0, IBM Inc., Armonk, NY, USA).

## Results

After applying the inclusion and exclusion criteria 5,952 patients from 142 hospitals were left for analysis. 2,468 (41.5%) of them had been intubated prior to admission. The GCS and intubation status distribution are shown in Table 1. While 97.4% of patients with GCS = 3 had been intubated at admission only 37 (1.1%) of 3,238 patients with an GCS ≥ 10 arrived intubated. In the group with a GCS 4–9 at admission only 54 of 274 (19.7%) had been intubated prior to admission, 80.3% were transferred without intubation.

**Table 1:** Distribution of GCS and intubated patients at admission

GCS	3	4	5	6	7	8	9	10	11	12	13	14	15
Cases %	2,440 41	26 0.4	21 0.4	40 0.7	40 0.7	55 0.9	92 1.5	119 2	130 2.2	195 3.3	340 5.7	616 10.3	1,838 30.9
Intubated %	2,377 97.4	15 57.7	8 38.1	11 27.5	6 15.0	6 10.9	8 8.7	4 3.4	1 0.8	3 1.5	5 1.5	7 1.1	17 0.9

Number of patients (cases) and percentages per GCS and number (percentage) of intubated patients at admission per GCS

GCS, Glasgow Coma Scale

Although guidelines exist for intubation of patients with GCS < 9 prior transportation [7, 20], our final analysis was conducted on patients presenting with GCS 4–9 at admission, only. We intentionally extended our GCS threshold for analysis to include GCS = 9 patients to enlarge our patient sample for a more powerful statistical statement and excluded patients with GCS = 3 because intubated patients are commonly sedated and predominantly present with this GCS, representing a significant confounder.

We compared the group of intubated (ITN; *n* = 2,468) vs. not-intubated (no-ITN; *n* = 3,484) traumatic brain injured patients at hospital admission. ITN patients were significantly younger (59.5 vs. 67.7 years; *p* < 0.001), had a lower ASA 3/4 prevalence (29.2% vs. 50.7%; *p* = 0.007), but higher ISS (24.3 vs. 21.7; *p* < 0.001). No statistical difference was found for AIS_Head_ between these two groups. In terms of vital parameters at admission and clinical hospital course ITN had a significant lower mean systolic blood pressure (SBP) (127 ± 18mmHg vs. 146 ± 28mmHg; *p* < 0.001), higher oxygen saturation (SAT) (98.6% vs. 96%; *p* < 0.001) at admission and more days (mean [IQR]) being intubated (4 days [1–14] vs. 1.5 days [0–9]; *p* = 0002). Differences also existed in lower mortality and sepsis rates in ITN patients (20.4% vs. 33.2%; *p* = 0.067 and 4.2% vs. 13.6%; *p* = 0.069; respectively) but did not reach statistical significance in this population. A comprehensive list of parameters is listed in Table 2.

**Table 2:** Patient and treatment characteristics if GCS on admission was 4–9

	Not intubated *N* = 220	Intubated *N* = 54	*p*-value
Age in years (SD)	67.7 (18.6)	59.5 (24.3)	**< 0.001**
Male gender	142 (64.5%)	42 (77.8%)	0.064
ASA 3/4 before injury	104 (50.7%)	14 (29.2%)	**0.007**
ISS (SD)	21.7 (7.3)	24.3 (9.5)	**< 0.001**
AIS_Head_			
AIS 3 AIS 4 AIS 5/6	43 (19.5%) 70 (31.8%) 107 (48.6%)	13 (24.1%) 12 (22.2%) 29 (53.7%)	0.369
*Parameters*,* management and complications during hospital treatment*			
Admission SBP in mmHg (SD)	146 (28)	127 (18)	**< 0.001**
Base excess (SD)	-1.1 (4.6)	-0.6 (4.8)	0.879
Coagulation INR (SD)	1.21 (0.48)	1.22 (0.56)	0.429
Oxygen saturation in % (SD)	96.0 (4.0)	98.6 (1.9)	**< 0.001**
ER intubation	84 (38.2%)	---	---
Acute cranial surgery	64 (42.4%)	10 (38.5%)	0.708
Cranial surgery	141 (64.1%)	34 (63.0%)	0.877
MOF	78 (37.1%)	18 (34.0%)	0.667
Sepsis	27 (13.6%)	2 (4.2%)	0.069
Mortality	73 (33.2%)	11 (20.4%)	0.067
LOI in median days [IQR]	1.5 [0–9]	4 [1–14]	**0.002**
LOICU in median days [IQR]	6.5 [3–16]	7 [3–19]	0.452
LOHS in median days [IQR]	12 [5–23]	13 [6–28]	0.440
Discharge to			
Home Rehabilitation Other hospital Any other	29 (19.7%) 66 (44.9%) 40 (27.2%) 12 (8.2%)	10 (23.3%) 20 (46.5%) 11 (25.6%) 2 (4.7%)	0.847
Functional outcome at discharge (GOS)			
Good recovery Moderate disability Severe disability Persistent vegetative state	38 (26.8%) 53 (37.3%) 43 (30.3%) 8 (5.6%)	14 (33.3%) 11 (26.2%) 12 (28.6%) 5 (11.9%)	0.323

Characteristics are shown for patients with an admission GCS 4–9 only and for intubated and not-intubated patients at the time of hospital admission

Percentages are shown for each characteristic within the “not intubated” or “intubated” subgroup. Number of patients evaluated for each characteristic may vary due to missing data

ER intubation is defined as intubation in the emergency room

Acute cranial surgery is defined as either acute cranial decompression in the ER (e.g. borehole, etc.) or immediate transport to the OR for cranial surgery

Cranial surgery is defined as any surgery to the head within hospital stay

Discharge destination and functional outcome are shown for surviving patients only

AIS, abbreviated injury scale; ASA, American Society of Anesthesiologists (ASA) physical status; ISS, injury severity scale; ER, emergency room; GCS, Glasgow Coma Scale; GOS, Glasgow Outcome Scale; INR, international normalized ratio; IQR, interquartile ratio; LOHS, length of hospital stay; LOI, length of intubation; LOICU, length of ICU stay; MOF, multiple organ failure; N, number of patients; OR, operating room; SBP, systolic blood pressure; SD, standard deviation

Because of the mortality difference between these two groups, we sought to determine whether we were able to identify significant independent risk factors for mortality, being the dependent variable in a multivariate logistic regression model among the 274 patients admitted to the ER with a GCS 4–9. The results are shown in Table 3.

**Table 3:** Multivariate logistic regression analysis with mortality as dependent variable

Predictor	OR	95% CI for OR	*p*-value
Age (reference: < 60 years) 60–69 70–79 80 and older	2.41 3.68 11.44	0.74–7.86 1.35–10.07 4.44–29.46	< 0.001 0.146 0.011 < 0.001
Pre-existing disease (ASA 3/4)	1.85	0.98–3.51	0.058
Coagulopathy	2.28	1.12–4.65	**0.023**
Head injury severity (AIS_Head_)			**0.002**
AIS_Head_ = 4	1.66	0.56–4.97	0.361
AIS_Head_ = 5/6	4.57	1.66–12.63	**0.003**
Relevant accompanying injury (AIS 2/3)	1.07	0.55–2.11	0.839
Intubated prior to admission	0.81	0.34–1.9	0.624

Total patients *N* = 274; GCS 4–9 on admission only

Nagelkerke’s R² = 0.384

Coagulopathy is defined as Quick’ value ≤ 60%, or PTT ≥ 40 s, or INR ≥ 1.4

Relevant accompanying injury is defined as any additional injury, besides head injuries, with AIS = 2 or 3

AIS, Abbreviated Injury Scale; ASA, Classification of American Society of Anesthesiologists; CI, confidence interval; OR, odds ratio;

Significant independent risk factors for mortality in patients being transferred with a GCS 4–9 upon admission to the ER were age older than 70 years (OR 3.68, *p* = 0.011) and 80 years (OR 11.44, *p* < 0.001), measurable coagulopathy upon admission (OR 2.28, *p* = 0.023) and AIS_Head_=5 or 6 (OR 4.57, *p* = 0.003). Accompanying injuries (AIS = 2 and 3) to other body regions had no significant effect on mortality. Being intubated prior to admission had a rather positive (OR = 0.81), but not significant (*p* = 0.62) effect on mortality. We report a Nagelkerke’s R² = 0.384 and thus a reasonable degree of explanatory power.

Out of 220 no-ITN patients admitted to the hospital a total of 84 patients (38.2%) required immediate intubation in the ER. For further analysis, whether withholding an intubation prior to transferal is possibly harmful, we first determined the GCS distribution of patients being intubated in the ER upon admission in the receiving hospital (ER ITN) and those not being intubated in the ER (ER no-ITN) **(**Table 4**)**. Within the ER ITN group an almost equal distribution of GCS 6–9 was found, whereas GCS 4 and 5 had a slightly lower prevalence in the ER intubated group.

**Table 4:** Distribution of patients with GCS 4–9 requiring emergency intubation, after being transferred without intubation (*n* = 220)

GCS	4	5	6	7	8	9	∑
ER ITN %	6 7.1	10 11.9	17 20.2	15 17.9	18 21.4	18 21.4	84
ER no-ITN %	5 3.7	3 2.2	12 8.8	19 14	31 22.8	66 48.5	136

Distribution of patients and percentages per GCS requiring emergency intubation in the ER (ER ITN) or being admitted without intubation in the ER (ER no-ITN)

ER, emergency room; GCS, Glasgow Coma Scale

We further compared the groups of ER ITN and ITN to identify differences in clinical outcome. As depicted in Table 5, later intubation after transfer resulted in a significant increase in prevalence of multiple organ failure (MOF) (53.7% vs. 34%; *p* = 0.025), sepsis (20.5% vs. 4.2%; *p* = 0.011) and mortality rate (38.1% vs. 20.4%; *p* = 0.028). In comparison to the aforementioned results in Table 2, the compared groups of ER ITN and ITN did not show significant differences in ISS and duration of intubation (LOI) anymore but changed to demonstrate significant differences in higher rates in cranial surgery, MOF, sepsis and mortality, as stated above.

**Table 5:** Management and clinical course of patients being intubated before (*n* = 54) and after (*n* = 84) inter-hospital transfer

	Intubated after admission (ER ITN) *N* = 84	Intubated prior to admission (ITN) *N* = 54	*p*-value
Age in years (SD)	67.2 (18.9)	59.5 (24.3)	**0.010**
Male gender	56 (66.7%)	42 (77.8%)	0.160
ASA 3/4 before injury	36 (42.9%)	14 (25.9%)	**0.043**
ISS (SD)	22.4 (7.2)	24.3 (9.5)	0.772^**#**^
AIS_Head_			
AIS 3 AIS 4 AIS 5/6	13 (15.5%) 21 (25.0%) 50 (59.5%)	13 (24.1%) 12 (22.2%) 29 (53.7%)	0.452
*Parameters*,* management and complications during hospital treatment*			
Admission SBP in mmHg (SD)	147 (27)	127 (18)	**< 0.001**
Base excess (SD)	-1.5 (5.2)	-0.6 (4.8)	0.945
Coagulation INR (SD)	1.30 (0.65)	1.22 (0.56)	0.767
Oxygen saturation in % (SD)	96.0 (4.5)	98.6 (1.9)	**< 0.001**
Acute cranial surgery	37 (66.1%)	10 (38.5%)	**0.019** ^**#**^
Cranial surgery	72 (85.7%)	34 (63.0%)	**0.002** ^**#**^
MOF	44 (53.7%)	18 (34.0%)	**0.025** ^**#**^
Sepsis	16 (20.5%)	2 (4.2%)	**0.011** ^**#**^
Mortality	32 (38.1%)	11 (20.4%)	**0.028** ^**#**^
LOI in days	6.5 [1–14]	4 [1–14]	0.601^**#**^
LOICU in days	10.5 [4–20]	7 [3–19]	0.528
LOHS in days	14 [6–26]	13 [6–28]	0.826
Discharge to			
Home Rehabilitation Other hospital Any other	9 (17.3%) 25 (48.1%) 17 (32.7%) 1 (1.9%)	10 (23.3%) 20 (46.5%) 11 (25.6%) 2 (4.7%)	0.709
Functional outcome at discharge (GOS)			
Good recovery Moderate disability Severe disability Persistent vegetative state	12 (23.5%) 14 (27.5%) 21 (41.2%) 4 (7.8%)	14 (33.3%) 11 (26.2%) 12 (28.6%) 5 (11.9%)	0.526

Characteristics are shown for patients with an initial admission GCS 4–9 only

Intubated after admission is defined as intubation in the emergency room

Acute cranial surgery is defined as either acute cranial decompression in the ER (e.g. borehole, etc.) or immediate transport to the OR for cranial surgery

Cranial surgery is defined as any surgery to the head within hospital stay

Discharge destination and functional outcome are shown for surviving patients only

LOI, LOICU and LOHS are shown in median and IQR

^**#**^ indicates change in significance compared to Table 2

AIS, abbreviated injury scale; ASA, American Society of Anesthesiologists (ASA) physical status; ISS, injury severity scale; ER, emergency room; GCS, Glasgow Coma Scale; GOS, Glasgow Outcome Scale; INR, international normalized ratio; IQR, interquartile ratio; LOHS, length of hospital stay; LOI, length of intubation; LOICU, length of ICU stay; MOF, multiple organ failure; N, number of patients; OR, operating room; SBP, systolic blood pressure; SD, standard deviation

## Discussion

In this retrospective analysis of adult patients suffering from at least a serious TBI and being transferred to a German level 1 or 2 trauma hospital, results suggest inferior outcome when intubation in GCS 4–9 is delayed until admission at the receiving hospital.

Endotracheal intubation is considered the gold standard in definitive airway protection, and patients with severe TBI are commonly intubated in the field by prehospital emergency medical personnel [4]. This treatment aims to prevent hypoxia, hypercapnia, and aspiration which could contribute to secondary injuries. In some health care systems, it is current practice to perform endotracheal intubation in the prehospital setting to secure the airway and permit controlled ventilation, as opposed to using basic maneuvers and adjuncts with supplemental oxygen [24]. Yet, the practice of prehospital intubation for airway management in TBI patients has produced conflicting outcome results in various studies, even suggesting implications on mortality [10–14, 25–28]. This is why some authors suggest not to strictly adhere to prehospital intubation in patients with moderate to severe TBI and reduced GCS, but rather individually weigh potential benefits and harms [12–14, 29]. 

Besides prehospital management of TBI patients, interhospital transfer of TBI patients is essential for patients being admitted to an ACTH but requiring specialized neurotrauma and/or neurocritical care. An increased transfer probability of TBI patients was associated with factors reflecting the number and severity of head injuries, a reduced GCS score and an increased New Injury Severity Score (NISS), as well as comorbidities in patients aged < 77 years [17] and on the receiving surgeon’s assessment and decision-making [19]. Several factors influencing their transfer decisions were identified, captured in six main themes under one overarching theme: “The chance of a favourable outcome”. These main themes were (A) “Establish TBI severity: Glasgow Coma Scale score and head CT”, (B) “Preinjury health status: comorbidity, functioning, and age”, (C) “Distance from ACTH to NTC: distance is time and time is brain”, (D) “Uncertainty and insecurity’” (E) “Capacity at NTC”, and (F) “Next of kin involvement” [19]. The above stated factors create a cohort of transferred patients that is different from the primarily admitted patients after prehospital treatment. Nevertheless, the underlying questions whether to intubate a patient with impaired consciousness prior transportation and the possibly associated effect on outcome remain valid and, to the best of our knowledge, have not been addressed before in these patients.

Although guidelines for transferring patients with TBI exist that patients with a GCS ≤ 8 or a significantly deteriorating conscious level should undergo tracheal intubation and mechanical ventilation [7, 20] we report a patient cohort with 80.3% arriving in the ER unintubated despite a recorded GCS ranging from 4 to 9. A relevant altered mental status and significant TBI can be assumed, because 38.2% of patients arriving without intubation required intubation upon arrival in the ER. We compared these initially transferred two groups for outcome-relevant differences but were not able to detect significant differences in mortality, MOF, sepsis, discharge destination or functional outcome at discharge. In our multivariate regression analysis model only age > 70 years, existing coagulopathy at hospital admission and AIS_Head_ ≥ 5 were significantly associated with increased mortality, while preexisting medical conditions, relevant accompanying injuries and intubation prior to admission had no significant effect on mortality in our patient cohort. In comparison, in patients with severe TBI various previously reported factors (e.g. age, coagulopathy, hypotension, hypoxemia, trauma scores and brain injury types) had shown to significantly contribute to mortality risk [30]. 

Because a relevant proportion (38.2%) of our transferred patients required intubation upon arrival in the ER we sought to determine whether omitting intubation prior to transportation might have had a detrimental effect on patient outcome. Therefore, we statistically compared the ER intubated patients (ER ITN) to the already intubated (ITN). These groups did not differ in injury severity, but ER ITN were significantly older and had more serious medical conditions (ASA 3/4). It has been shown that in major traumatic brain injury out-of-hospital hypotension and hypoxia were associated with significantly increased mortality [31, 32]. In our study, intubated and not-intubated patients at admission did significantly differ in mean SBP and oxygen saturation, but the mean value + SD were well above the critical values of 90mmHg (SBP) and 90% (SAT).

The necessity of acute cranial surgery in our collective of transferred patients was 38.5% (ITN) and 42.4% (no-ITN) but increased to 66.1% in patients requiring intubation in the ER upon arrival (ER ITN). Our initial rate in transferred patients is comparable to a published prevalence of decompressive craniectomy (31.4%) in transferred TBI patients [33]. The increased prevalence in the ER ITN group might be a result of surgeon’s judgement to emergency surgery and therefore anticipated intubation upon arrival.

The major results in our study are the significant differences in MOF, sepsis and mortality rates when transferred patients arrive at the ER with an indication for intubation. In contrast to our first comparison between all intubated and non-intubated transferred patients the focus on possibly “delayed” intubated (ER ITN) patients revealed higher prevalence in MOF (53.7% vs. 34%; *p* = 0.025), sepsis (20.5% vs. 4.2%; *p* = 0.011) and mortality rate (38.1% vs. 20.4%; *p* = 0.028). In comparison to other studies our results lay within the published prevalence of up to 80–90% organ failure [34, 35], 3.3% − 11% sepsis and 9% − 36% mortality rates [36, 37]. 

## Limitations

There are multiple limitations to this study, mostly due to its retrospective nature. All hospitals participating in the TR-DGU submit to regular audits and sample tests are performed to ensure data quality. However, the documentation’s validity is not controlled by external monitoring as in prospective trials [38]. Although mainly Level 1 and 2 trauma centers contribute to this database, we cannot comment on locally implemented protocols in trauma care, especially care for severe TBI patients. However, our patient cohort received care in German hospitals where training in ATLS^®^ courses and protocols has been established since 2003 [39]. 

Since the TR-DGU database is based on data entered by the receiving hospitals we cannot comment on possible deterioration (e.g. GCS, vital signs) during patient transport. Unfortunately, it is not possible to link patient data of the transferring and accepting hospital within the TR-DGU database and therefore we are not able to state on the initial presentation and treatment of patients in the transferring hospitals or their clinical path during transport. Therefore, we might have included patients that had a significant clinical deterioration during transport, consequentially requiring intubation at the ensuing hospital admission, but who had no indication for invasive airway management prior transportation.

Importantly, the registry does not contain peritransfer variables such as prehospital treatment before admission to the transferring hospital, time from accident to transfer initiation, transfer duration or transfer logistics. Thus, our results must be judged accordingly. Especially, since prehospital and interhospital treatment as well as emergency personnel during transport and transfer modality (e.g. helicopter vs. ground based, physician staffed vs. paramedic staffed) differs between countries, our results from German hospitals cannot be readily transferred to other health care systems.

Finally, the findings of our investigation rely on the evaluation of consciousness levels represented in sum score GCS values. In clinical practice, the overall reliability of the GCS seems to be adequate, but the reliability of the sum score is less than that of the components of the GCS [40] with especially the combination of pupil reactivity and GCS motor component outmatching the predictive accuracy of GCS alone [41]. 

## Conclusion

In this retrospective analysis of interhospital transferred multiply injured patients suffering from at least a serious TBI, our results suggest inferior outcome when intubation in patients with GCS 4–9 is needed during admission at the receiving hospital. Postponed intubation seems to possibly result in higher prevalences in MOF (53.7% vs. 34%; *p* = 0.025), sepsis (20.5% vs. 4.2%; *p* = 0.011) and mortality rate (38.1% vs. 20.4%; *p* = 0.028) when compared to severely brain injured patients admitted already intubated at the receiving hospital. Based on our results, further research is warranted to scrutinize optimal airway management for interhospital transfer of TBI patients with a reduced level of consciousness. 

## Data Availability

The data that support the findings of this study are available from the AUC—Academy for Trauma Surgery (AUC—Akademie der Unfallchirurgie GmbH), but restrictions apply to the availability of these data, which were used under license for the current study, and so are not publicly available. Data are however available from the corresponding author J.B. upon reasonable request and with permission of AUC (AUC—Akademie der Unfallchirurgie GmbH, Emil-Riedel-Straße 5, 80538 München, Deutschland).
